# Enzyme-catalyzed Ag Growth on Au Nanoparticle-assembled Structure for Highly Sensitive Colorimetric Immunoassay

**DOI:** 10.1038/s41598-018-24664-w

**Published:** 2018-04-19

**Authors:** Xuan-Hung Pham, Eunil Hahm, Tae Han Kim, Hyung-Mo Kim, Sang Hun Lee, Yoon-Sik Lee, Dae Hong Jeong, Bong-Hyun Jun

**Affiliations:** 1 Department of Bioscience and Biotechnology, Konkuk University, Seoul, 143-701 Republic of Korea; 2 Department of Chemistry Education, Seoul National University, Seoul, 151-742 Republic of Korea; 3 School of Chemical and Biological Engineering, Seoul National University, Seoul, 151-742 Republic of Korea

## Abstract

We have developed a sensitive colorimetric immunoassay with broad dynamic range using enzyme-catalyzed Ag growth on gold nanoparticle (NP)-assembled silica (SiO_2_@Au@Ag). To reduce Ag^+^ ion content and promote Ag growth on the assembled Au NPs, alkaline phosphatase (AP)-based enzymatic amplification was incorporated, which considerably increased the colorimetric read-out. As a model study, sandwich enzyme-linked immunosorbent assay (ELISA) was used to quantify target IgG. The immune complexes capture the Ab-IgG-AP-labeled detection Ab and trigger the enzyme-catalyzed reaction to convert 2-phospho-L-ascorbic acid to ascorbic acid in the presence of the target IgG. Ascorbic acid reduced Ag^+^ to Ag, which formed Ag shells on the surface of SiO_2_@Au and enhanced the absorbance of the SiO_2_@Au@Ag solution. Plasmonic immunoassay showed a significant linear relationship between absorbance and the logarithm of IgG concentration in the range of ca. 7 × 10^−13^ M to 7 × 10^−11^ M. The detection limit was at 1.4 × 10^−13^ M, which is several hundred folds higher than that of any conventional colorimetric immunoassay. Thus, our novel approach of signal-amplification can be used for highly sensitive *in vitro* diagnostics and detection of target proteins with the naked eye without using any sophisticated instrument.

## Introduction

Immunological assays in medicine are one of the most prominent analytical techniques for early diagnosis of diseases and monitoring of treatment efficacy. Extensive research efforts have been directed at improving the sensitivity of these methods by combining immunological techniques with various analytical techniques such as surface plasmon resonance^1,2^, quartz crystal microbalance^3^, surface-enhanced Raman spectroscopy^4^, fluorescence spectroscopy^5,6^, electrochemistry^7–13^, chemiluminescence^14^, and colorimetric assay^15,16^. Among these, the enzyme-linked immunosorbent assay (ELISA) is a popular and low-cost colorimetric immunoassay for assaying clinically salient target molecules^17–20^ because of its simplicity and practical applicability, which allows rapid/direct readout with the naked eye^21–27^. However, conventional ELISA is limited to a narrow dynamic range for quantitative detection^28^, with picomolar level detection limit, which renders them incapable of detecting scarce proteins in body fluids or tissues^29^. As a result, methods for enhancing ELISA sensitivity via amplification of enzyme-mediated signals are being actively studied.

Recently, ELISA with ultra-high sensitive nanomaterial-based signal amplification has attracted considerable attention^30^. Since nanomaterials possess features such as catalytic activity, conductivity, and biocompatibility, they can be utilized to accelerate signal transduction and amplification in ELISA. Most importantly, nanoscale materials can function as sensors with resolution of single-molecule detection^31^. Gold nanoparticles (AuNPs) have been widely utilized in bio-analytics due to their superb biocompatibility, and ease of surface modification and preparation^32^. A variety of highly sensitive monodispersed or aggregated AuNP-based immunoassays have been reported^15,33–36^. However, most of these immunoassays cannot precisely quantify the concentration of target molecules because of their narrow linear range of target detection^33^. Therefore, development of highly sensitive and quantitative AuNP-based immunoassays is considerably challenging.

Bimetallic Au-Ag NPs can provide an alternative solution as localized surface plasmon resonance (LSPR) of Au-Ag NPs can enhance the strength of electromagnetic field around NP surface. In addition, Au-Ag alloy NPs offer several distinctive advantages: 1) improved plasmonic efficiency; 2) higher refractive index sensitivities exclusively from Ag; and 3) long-term stability and biocompatibility from Au^37^. In particular, the optical properties of Au-Ag NPs can be tuned by manipulating the growth of Ag shell thickness on Au NPs^38–42^. Recently, assembly of Au and/or Ag NPs on a silica NP template was reported by several groups^41,43–53^. Higher LSPR was observed from Au and/or Ag NPs of controlled density, in which the gaps between the NPs were adjusted, than from either Au or Ag NP alone^54^.

In this study, we have controlled Ag deposition on AuNP surface, assembled on silica NPs by an enzyme-catalyzed reaction. Results showed amplified ELISA signals and improvement in the sensitivity of colorimetric immunoassay. In our model study, IgG concentration was determined from color change, and absorbance intensity was detected by the formation of Ag shells on the surface of Au NPs. The plasmonic immunoassay showed a significantly linear relationship between absorbance and logarithm of IgG concentration with thousand folds higher sensitivity than that of any conventional colorimetric immunoassay.

## Results and Discussion

**Figure 1:**
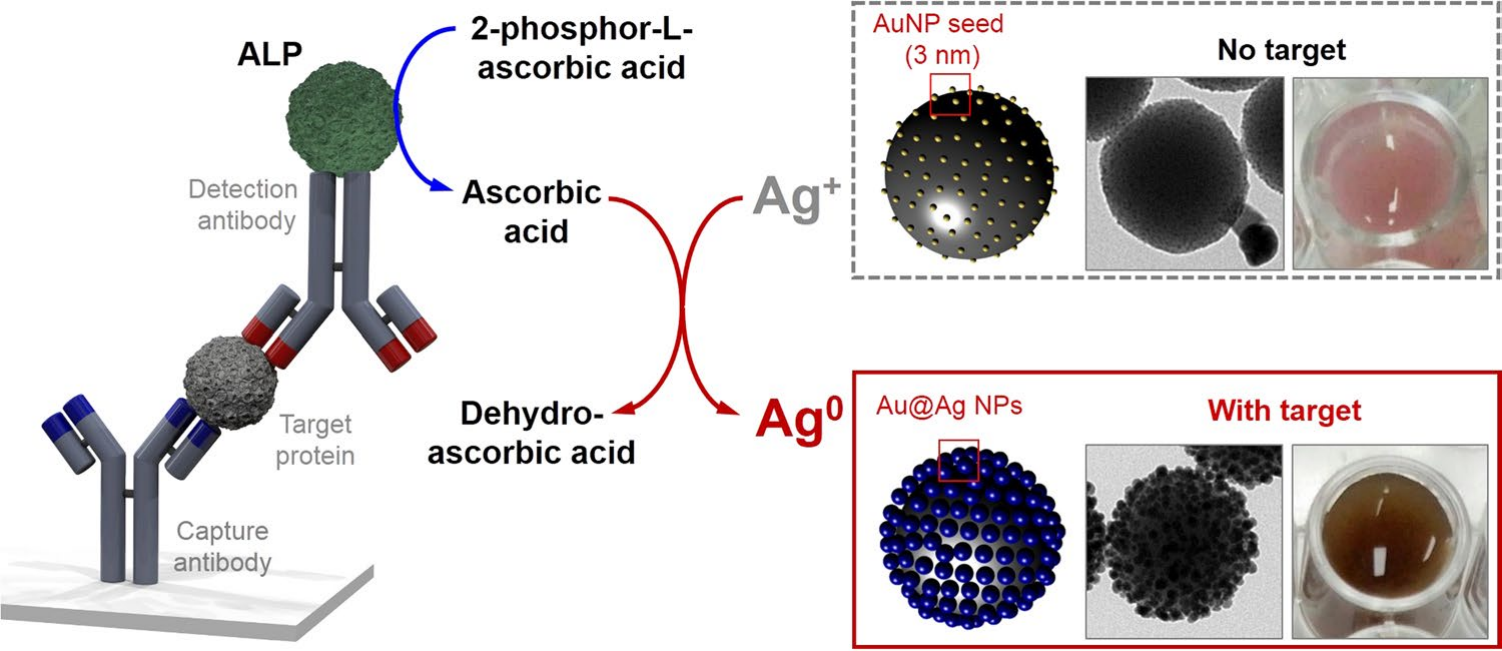
Schematic representation of enzyme-catalyzed Ag growth on Au nanoparticle-assembled structures. In the presence of target IgG, the immune complex Ab_1_-IgG-Ab_2_, which contains alkaline phosphatase (AP), triggers enzyme-catalyzed conversion of 2-phospho-L-ascorbic acid to ascorbic acid. The ascorbic acid reduces AgNO_3_ to Ag metal which can be deposited on the surface of AuNPs on silica NPs. As Ag deposition on the surface of SiO_2_@Au results in strong absorbance, significant colorimetric changes can be observed, allowing for highly sensitive colorimetric immunoassay.

The plasmonic-based colorimetric immunoassay (Fig. 1) caused a strong colorimetric change by combining two features – enzyme-mediated amplification of Ag^+^ ion reduction and promotion of Ag growth on the assembled AuNP structure (SiO_2_@Au@Ag). As a model study, rabbit IgG (antigen) was utilized as a target for the immunoassay. Polyclonal goat anti-rabbit IgG (capture antibody, Ab1), which was used as a platform to capture the rabbit IgG, was immobilized on a 96-well microplate. Subsequently, polyclonal AP-conjugated goat anti-rabbit IgG (detection antibody, Ab_2_) was used as a tracer antibody in the plasmonic-based colorimetric immunoassay. In the presence of the target IgG, the immune complex of Ab_1_-IgG-Ab_2_, which contained alkaline phosphatase (AP), was able to trigger enzyme-catalyzed conversion of 2-phospho-L-ascorbic acid to ascorbic acid. The produced ascorbic acid reduced AgNO_3_ to Ag metal, which was deposited on the surface of AuNPs on silica NPs (SiO_2_@Au). The deposition shifted the ultraviolet-visible (UV-Vis) spectra towards 430 nm, which coincided with the absorbance peak of Ag. The shift in absorbance was proportional to the IgG concentration in the sample, which was quantified by monitoring the intensity of absorbance at 430 nm.

One prerequisite for developing effective plasmonic-based colorimetric immunoassay is the coating of Ag metal on the SiO_2_@Au surface in the presence of the ascorbic acid reduction system. To demonstrate this, SiO_2_@Au was prepared by immobilizing Au NPs on aminated silica NPs (ca. 150 nm in diameter). Colloidal AuNPs (2–3 nm) were prepared by reducing HAuCl_4_ with tetrakis (hydroxymethyl) phosphonium chloride (THPC) according to the method reported by Duff *et al*. with slight modification^55^. The AuNPs were immobilized on the aminated silica NPs by gentle shaking. Figure S1 shows the assembly of ca 2,300 AuNPs on the surface of the aminated silica NPs. Selective formation of Ag shells on the surface of Au NPs on silica occurred when AgNO_3_ were reduced in the presence of ascorbic acid and polyvinylpyrrolidone (PVP). The thickness of AgNPs was adjusted by the ascorbic acid concentration in the solution. AgNO_3_ concentration was maintained at 10 mM, whereas that of ascorbic acid varied from 2 to 100 µM. The amount of SiO_2_@AuNPs was fixed at 100 µg.

**Figure 2:**
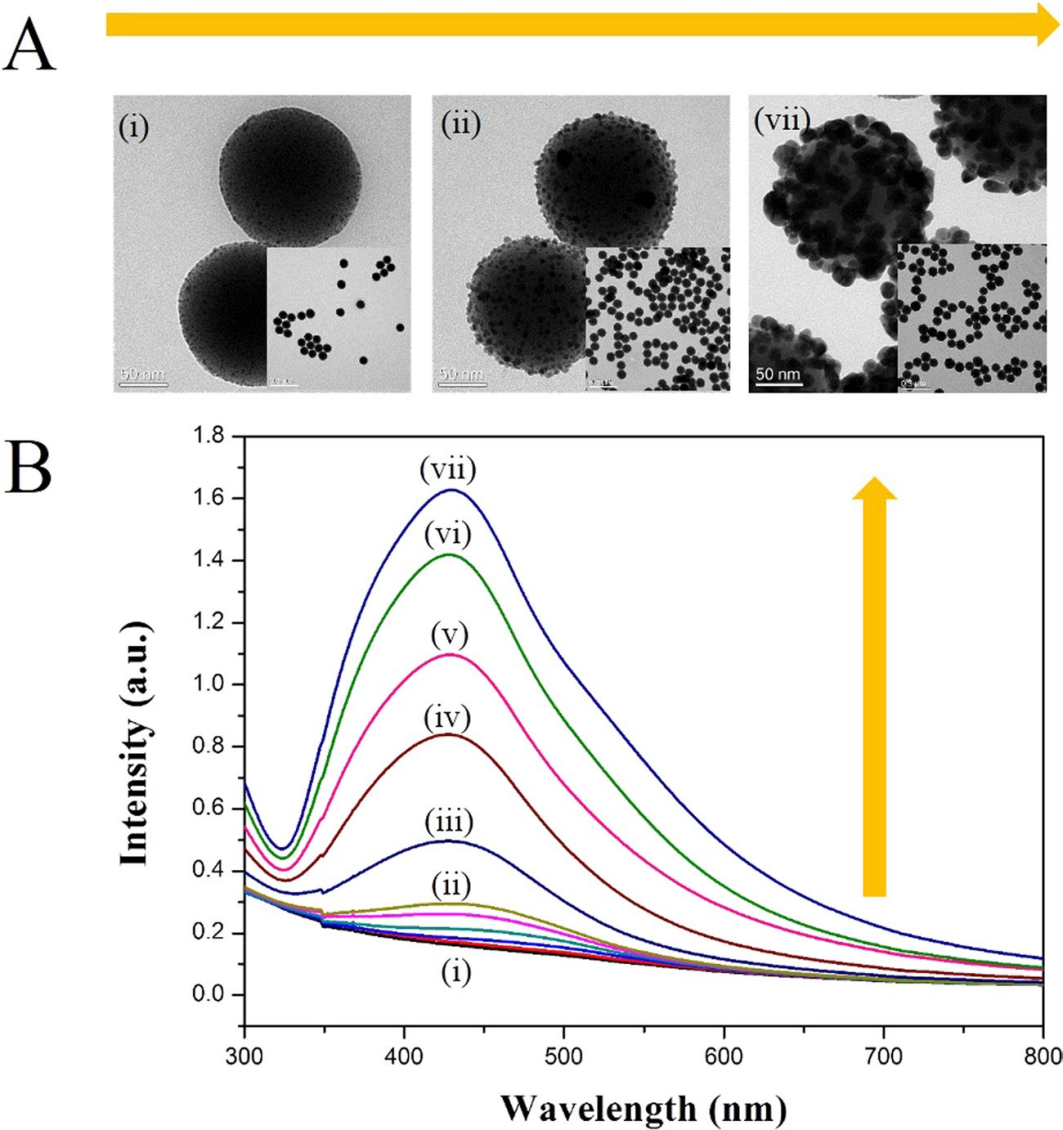
(**A**) Transmission electron microscopy (TEM) images and (**B**) optical properties of SiO_2_@Au@Ag solution at different concentrations of ascorbic acid: (i) 0 µM, (ii) 10 µM, (iii) 20 µM, (iv) 40 µM, (v) 60 µM, (vi) 80 µM, and (vii) 100 µM. Measurement values for SiO_2_@Au and AgNO_3_ are 100 µg and 1 nmol, respectively.

Typical transmission electron microscopy (TEM) images are shown in Figs 2A and S2. The size of Au@AgNPs on the surface of the silica NPs increased with the concentration of ascorbic acid from 2 to 100 µM. UV-Vis spectra of the resulting SiO_2_@Au@Ag solution are shown in Fig. 2, which match well with their corresponding TEM images. Owing to low absorbance of the 3 nm-sized AuNPs, SiO_2_@AuNPs did not show the typical UV peak of AuNPs at 500–520 nm^55^. A broad plasmonic adsorption peak was observed between 320 nm and 700 nm, with its maximum at ca. 430 nm, after deposition of Ag metal on SiO_2_@AuNPs, which can be attributed to the formation of Ag shells on the surface of SiO_2_@Au NPs^56–58^. In addition, the intensities of peaks at 430 nm (Ag) and 510 nm (Au) were proportional to the concentration of ascorbic acid across a broad range (2, 4, 6, 8, 10, 20, 40, 60, 80, and 100 µM), indicating proportional growth of the Ag shells on SiO_2_@Au@Ag (Figs 2B and S3)^58,59^. The appearance of a new absorbance peak of SiO_2_@Au@Ag at 430 nm could be explained by the positive effect of ascorbic acid-induced Ag shell coating on the SiO_2_@Au surface.

To investigate the functionality of the plasmonic-based colorimetric immunoassay, AP-conjugated goat anti-rabbit IgG was utilized as the detection antibody. First, we determined the correlation in optical properties between the immunocomplex (Ab_1_, Ab_2_ and IgG) and 2-phospho-L-ascorbic acid concentration (Fig. S4). Three sets of experiments were conducted with varying concentrations of 2-phospho-L-ascorbic acid (up to 16 mM). As shown in Figure S4 (a, top), the wells in the top row were incubated with Ab_2_, in the middle row with IgG, and the bottom row with both IgG and Ab_2_. In the absence of either IgG or Ab_2_, the resulting SiO_2_@Au@Ag solution showed no significant changes in color regardless of the 2-phospho-L-ascorbic acid concentration. In contrast, the colors of the resulting SiO_2_@Au@Ag containing IgG and Ab_2_ significantly changed from varying degrees of yellow to deep brown, indicating growth of Ag on the surface of SiO_2_@Au to generate SiO_2_@Au@Ag. The intensity of the SiO_2_@Au@Ag absorbance increased with 2-phospho-L-ascorbic acid concentration and achieved its highest value at 12 mM (Fig. S4b,c). The increase in absorption intensity in our assay can be attributed to the enzyme-based catalytic reaction of AP conjugated with Ab_2_.

We also investigated the effect of blocking duration with bovine serum albumin (BSA) on the absorbance signal of SiO_2_@Au@Ag. The microplate was incubated with 1% BSA to prevent nonspecific adsorption and the results are shown in Fig. S5. The background signal of SiO_2_@Au@Ag decreased slightly from 0.290 ± 0.042 to 0.232 ± 0.035 after incubating with BSA for 30 minutes. Longer incubation time with BSA did not improve this prevention effect but decreased the absorbance intensity of IgG (incubation of 120 minutes).

Next, we investigated the effect of enzymatic incubation time on absorbance intensity (Fig. S6). The absorbance intensity of the SiO_2_@Au@Ag solution was proportional to the incubation time and attained its highest value at 40 minutes. This indicates that the enzyme AP was able to efficiently catalyze and transfer 2-phospho-L-ascorbic acid to L-ascorbic acid in our assay. In addition, the seed amount of SiO_2_@Au can be a critical factor for enhancing the absorbance properties of SiO_2_@Au@Ag. Subsequently, we studied the effect of SiO_2_@Au loading amount on the growth of SiO_2_@Au@Au. The solution color changed from dark grey to light yellow with increase in the concentration of SiO_2_@Au from 1 mg/mL to 5 mg/mL. The absorbance intensity of SiO_2_@Au@Ag increased with SiO_2_@Au concentration. The SiO_2_@Au@Ag synthesized at 4 mg/mL showed the highest value of absorbance intensity as shown in Fig. S7.

To reduce the time required for performing an ELISA immunoassay, we attempted to combine the enzyme reaction and ascorbic acid-induced Ag NP deposition into one step, hereafter referred to as the “combination” step. In contrast, the term “separation” indicates that the enzyme reaction and ascorbic acid-induced Ag NP deposition were separated into two steps. The IgG concentration was fixed at 4 × 10^−10^ M. As a result, the absorbance intensity of the SiO_2_@Au@Ag in “combination” technique decreased to ~45% compared to that in “separation technique (Fig. S8). For comparing the combination and separation techniques, we changed IgG concentration from 7 × 10^−16^ to 7 × 10^−9^ M. The absorbance signal was not significantly different at high IgG concentration because the enzyme in solution was sufficiently saturated to catalyze the further conversion of all 2-phospho-L-ascorbic acid to L-ascorbic acid. The absorbance signal of the combination sample increased gradually when the IgG concentration ranged from of 4 × 10^−11^ to 7 × 10^−10^ M because of the gradual conversion of 2-phospho-L-ascorbic acid to L-ascorbic acid. In contrast, the disposable availability of L-ascorbic acid in the enzyme reaction step resulted in a sharp increase in absorbance signal in the separation method. Furthermore, we compared the effect of SiO_2_@Au concentration on the resulting absorbance intensity using this immunoassay. The absorbance signal was not significantly different at high IgG concentration because the enzyme in solution was amply saturated to support further conversion of all 2-phospho-L-ascorbic acid to L-ascorbic acid. The absorbance signal of the “combination” sample increased gradually when the IgG concentration ranged from 4 × 10^−11^ to 7 × 10^−10^ M because of the gradual conversion of 2-phospho-L-ascorbic acid to L-ascorbic acid. In contrast, the presence of L-ascorbic acid in the previous adjacent enzyme reaction step caused a sharp increase in absorbance signal in the “separation” method. Furthermore, the effect of SiO_2_@Au concentration on the resulting absorbance intensity was investigated. The absorbance intensity of SiO_2_@Au@Ag in the “combination” method increased gradually with the logarithm of IgG concentration and ranged from 7 × 10^−11^ M to 7 × 10^−9^ M. On the contrary, the absorbance intensity of SiO_2_@Au@Ag in the “separation” method increased sharply with the logarithm of IgG concentration and ranged from 7 × 10^−13^ M to 7 × 10^−11^ M because of the presence of higher concentration of L-ascorbic acid in the enzyme reaction step as mentioned previously. From these results, we concluded that the gradual increase in the absorbance intensity of the “combination” is possibly due to the partial inhibitory effect of Ag^+^ ions on AP activity. The limit of detection (LOD) and limit of quantification of the “separation” method were 0.02 ng/mL and 0.69 ng/mL, respectively. In contrast, the LOD and LOQ of the “combination” method were 0.22 ng/mL and 0.72 ng/mL.

**Figure 3:**
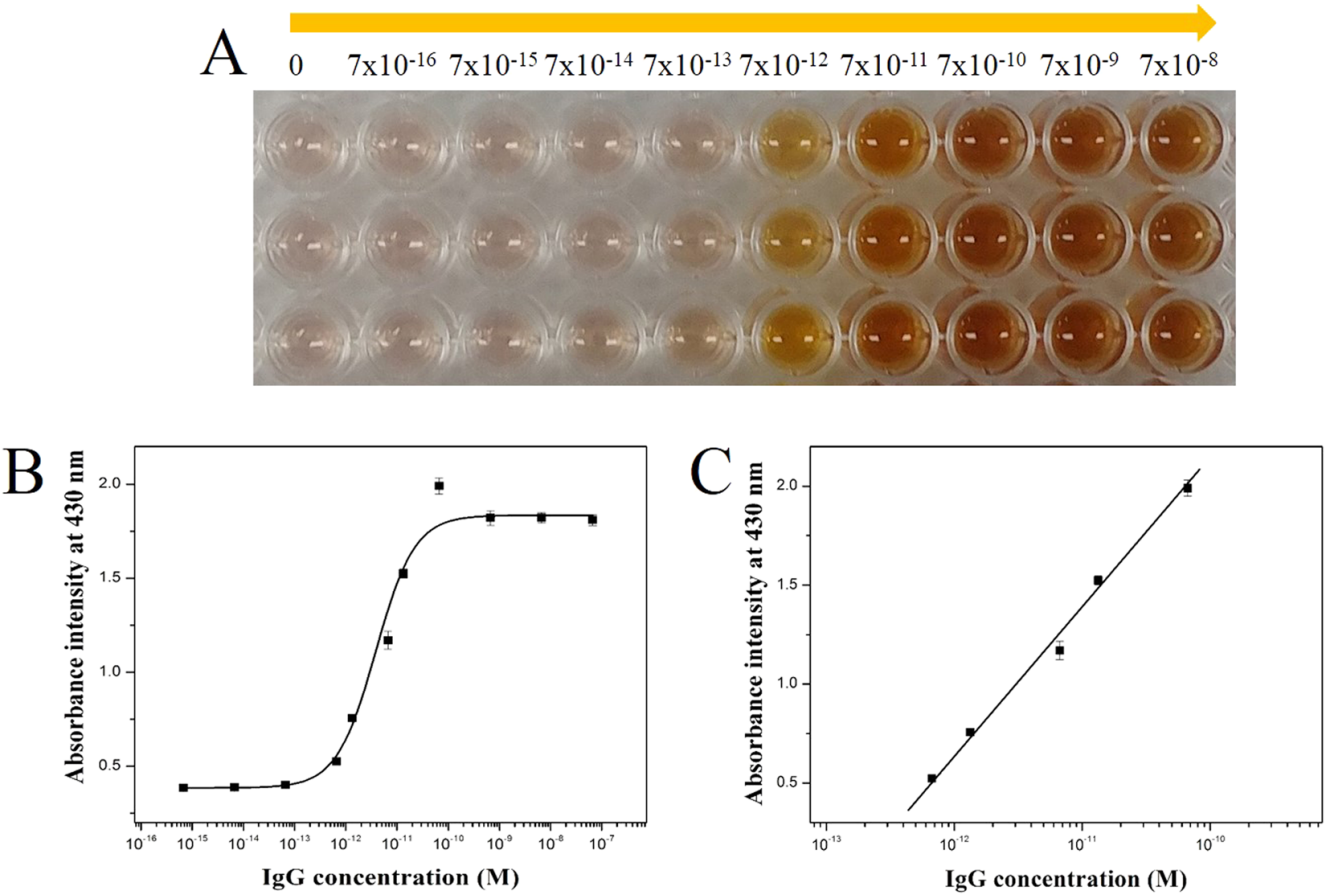
(**A**) Color change plot, (**B**) the corresponding UV-Vis absorbance intensity plot, and (**C**) dynamic range of plasmonic enzyme-linked immunosorbent assay with different concentrations of IgG in the range of 7 × 10^-16^ to 7 × 10^-8^ M. Inset shows the limit of detection of plasmonic-based colorimetric immunoassay. Error bars show the standard deviation of three independent measurements.

The absorbance intensity of the SiO_2_@Au@Ag solution at various IgG concentrations was obtained after optimizing the assay conditions. Figure 3A depicts the colors of the SiO_2_@Au@Ag solutions after enzyme-induced colorimetric enhancement. The solution color showed a dramatic change from light pink to deep brown when the concentration of IgG increased from 7 × 10^−16^ to 7 × 10^−7^ M, which implied that ascorbic acid was produced from 2-phospho-L-ascorbic acid in the solution by anti-rabbit IgG-conjugated AP, which was able to reduce Ag^+^ ion to Ag metal on the surface of SiO_2_@Au to generate SiO_2_@Au@Ag. The absorbance intensities of the reaction samples containing SiO_2_@Au@Ag are depicted in Fig. 3B. The absorbance intensity at 430 nm exhibited a slight change when the concentration of IgG was below 7 × 10^−14^ M. Insufficient amounts of Ab_2_ might have resulted in negligible ascorbic acid production in the solution, causing low levels of Ag metal deposition on the surface of SiO2@Au. On the contrary, IgG concentration > 7 × 10^−13^ M showed significant absorbance intensity of SiO_2_@Au@Ag at 430 nm. The absorbance intensity increased significantly with IgG concentration because of the growth of Ag on the surface of SiO_2_@Au (Fig. 3B). A logistic curve-fitting was utilized for calibration. A logistic curve relationship between the absorbance and the logarithm of IgG concentration from 7 × 10^−13^ M to 7 × 10^−11^ M could be fitted onto the experimental data. The LOD was 1.4 × 10^−13^ M (0.021 ng/mL). The relative standard deviation of our samples ranged from 0.5 to 9.6%. The detection limit of our technique showed values that were hundred folds higher than that of conventional ELISA. The LOD of our SiO_2_@Au-based method was 10-fold lower than that of Ag NP-based plasmonic ELISA^30^.

We determined the recovery to validate our method for detecting IgG concentration. We prepared two IgG samples of 3.34 × 10^−10^ M and 6.67 × 10^−10^ M concentration and used the logistic fitting curve to calculate the IgG concentration in the sample. Results showed that the IgG concentrations obtained were 3.56 ± 0.03 × 10^−10^ and 6.15 ± 0.03 × 10^−10^; which correspond to 106.8 ± 1.0% and 92.2 ± 1.6% recovery, respectively. Therefore, the average recovery is 99.5%.

## Conclusion

We have developed a novel plasmonic colorimetric immunoassay for detecting target proteins with a detection limit of 1.4 × 10^−13^ M. This assay features a dynamic range that is hundred times higher than that of the traditional enzyme-based colorimetric immunoassay, which was achieved by utilizing an AP-conjugated detection antibody to catalyze the conversion of 2-phospho-L-ascorbic acid to ascorbic acid. The presence of ascorbic acid induced the reduction of Ag^+^ ion to Ag metal on the surface of SiO_2_@Au, which enhanced the absorbance peak at 430 nm. As a result, the absorbance intensity of the SiO_2_@Au@Ag was linearly proportional to the logarithm of IgG concentration in the range from 7 × 10^−13^ M to 7 × 10^−11^ M. Our plasmonic colorimetric immunoassay is highly sensitive and covers a wide dynamic range. Therefore, it can be used for *in vitro* diagnostics for the detection of target proteins with the naked eye.

## Methods

### Materials

Tetraethylorthosilicate (TEOS), 3-aminopropyltriethoxysilane (APTS), silver nitrate (AgNO_3_), Tetrakis (hydroxymethyl) phosphonium chloride (THPC), gold (III) chloride trihydrate (HAuCl_4_), ascorbic acid (AA), polyvinylpyrrolidone (PVP), PBS buffer pH 7.4 tablet, bicarbonate buffer pH 8.5, Tween 20, 2-phospho-L-ascorbic acid, anti-rabbit IgG produced in goat (R2004), IgG from rabbit serum (I5006), anti-rabbit IgG-conjugated alkaline phosphatase antibody produced in goat (A3687), and anti-PSA were purchased from Sigma-Aldrich (St. Louis, MO, USA) and used without further purification. Ethyl alcohol (EtOH) and aqueous ammonium hydroxide (NH_4_OH, 27%) were purchased from Daejung (Siheung, Korea). F96 Maxisorp Nunc-Immuno microplate was purchased from Thermo Fisher Scientific (Roskilde, Denmark).

### Preparation of Au NPs assembled silica nanoparticles (SiO_2_@Au NPs)

Approximately 150 nm-sized silica NPs were prepared using Stöber’s method^60^. The surface of the aminated silica NPs were modified by amino groups using APTS and a previous method^56,57^. The colloidal Au NPs were prepared by reducing AuCl3 using THPC. THPC-capped Au NPs were prepared by the method reported by Duff *et al*^55,57^. Au NPs (1 mM, 10 mL) were modified on the surface of aminated SiO2 solution (1 mg/mL, 1 mL) by mixing and incubating them in a shaker overnight^50^. Au NP-embedded silica NPs were obtained by centrifugation and washed several times with EtOH to remove unbound Au NPs. The resulting SiO_2_@Au NPs were redispersed in absolute EtOH to obtain 1 mg/mL SiO_2_@Au NPs solution^56,57^.

### Preparation of SiO_2_@Au@Ag NPs

Au-Ag core-shell NPs were prepared in an aqueous medium by reducing and depositing Ag source with ascorbic acid on gold NPs in the presence of PVP. Briefly, 100 µg SiO_2_@Au NPs (100 µL) were dispersed in 0.7 mL PVP (1 mg/mL). AgNO_3_ (10 mM, 100 µL) was added to the solution, followed by the addition of 10 mM ascorbic acid (100 µL). This solution was incubated for 1 h to reduce Ag^+^ ion to Ag metal. The resulting SiO_2_@Au@Ag NPs were obtained by centrifugation at 8,500 rpm for 15 mins and washed several times with EtOH to remove excess reagent. The SiO_2_@Au@Ag NPs were re-dispersed in 1 mL absolute EtOH^56,57^.

### Procedure for detection of rabbit IgG

One hundred microliters of rabbit anti-IgG solution (10 µg/mL) was immobilized overnight in a 96-well microplate. After washing the microplate thrice with PBS containing 0.1% Tween 20 (PBST), it was blocked using 300 μL of 1% BSA for 30 minutes to prevent nonspecific adsorption. Then, rabbit IgG of various concentrations (100 µL) were injected into the microplate and incubated at 37 °C for 1 h for antibody-antigen interaction, followed by rinsing each well thrice with PBST. Next, AP conjugated anti-rabbit IgG (100 µL) was added to each well and incubated at 37 °C for 40 minutes, followed by washing with PBST.

One hundred microliters of 2-phospho-L-ascorbic acid (substrate) in a bicarbonate buffer (pH 8.5), 100 µL of SiO_2_@Au (1 mg/mL), and 100 µL of 10 mM AgNO_3_ in PVP (1 mg/mL) were simultaneously injected into the microplate and incubated at room temperature for 30 minutes. The prepared sample was diluted 10 times and its UV-Vis absorption spectra at 430 nm were measured.

## Electronic supplementary material

Supporting Information
