# Supplementary material for: Enzyme-catalyzed Ag Growth on Au Nanoparticle-assembled Structure for Highly Sensitive Colorimetric Immunoassay
Source: Sci Rep. 2018 Apr 19;8:6290. doi: 10.1038/s41598-018-24664-w (PMC5908853; doi:10.1038/s41598-018-24664-w)
Supplement: Supplementary file 1 — Supporting Information [file 41598_2018_24664_MOESM1_ESM.doc]

**Enzyme-catalyzed Ag Growth on Au Nanoparticle-assembled Structure for Highly Sensitive Colorimetric Immunoassay**

Xuan-Hung Pham,^a^ Eunil Hahm,^a^ Tae Han Kim,^a^ Hyung-Mo Kim,^a^ Sang Hun Lee,^c^ Yoon-Sik Lee,^c^ Dae Hong Jeong^b^ and Bong-Hyun Jun^a^*

*^a^ Department of Bioscience and Biotechnology, Konkuk University, Seoul 143-701, Republic of Korea.*

*^b^ Department of Chemistry Education, Seoul National University, Seoul 151-742, Republic of Korea.*

*^c^ School of Chemical and Biological Engineering, Seoul National University, Seoul 151-742, Republic of Korea*

**Name of Corresponding Author:** Bong-Hyun Jun, Ph.D.

Tel.: +82-2-450-0521, Fax: +82-2-3437-1977, E-mail: bjun@konkuk.ac.kr (B.-H. Jun)

**Keywords:** silica template, Au-Ag alloy, colorimetric immunoassay, IgG detection.


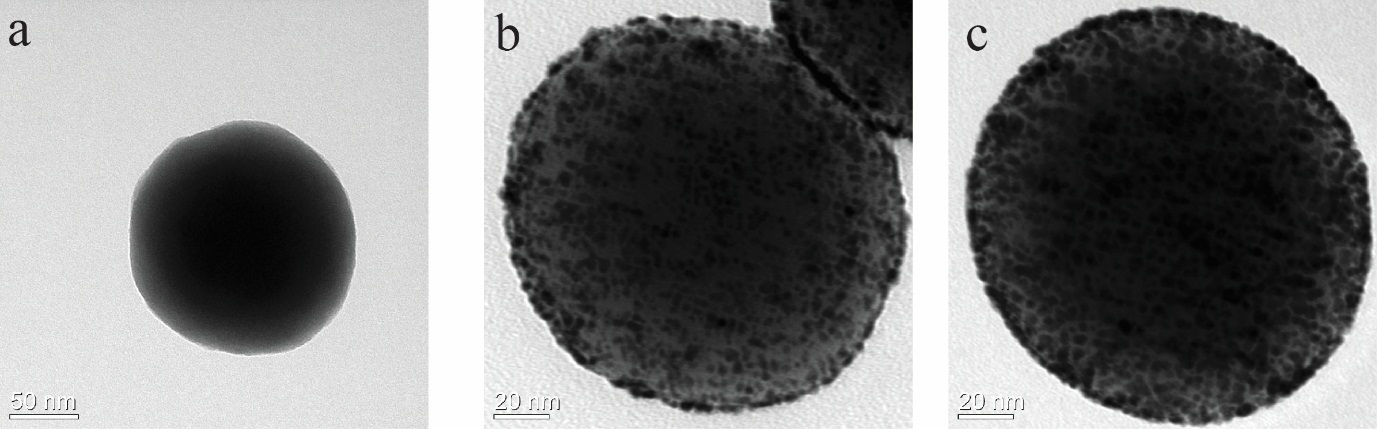


Figure S1. Transmission electron microscopy (TEM) images of (a) SiO_2_ NP and SiO_2_@Au NP with SiO_2_ modified with (b) NH_2_ groups (APTS) and (c) SH groups (MPTS).


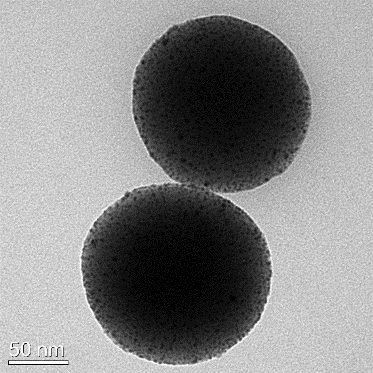

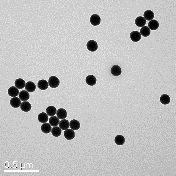

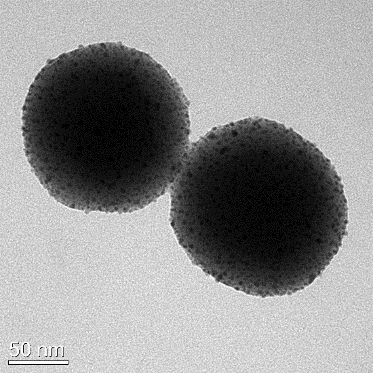

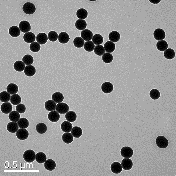

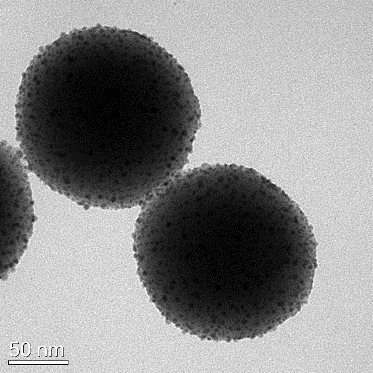

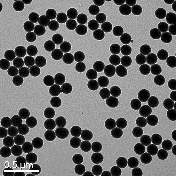

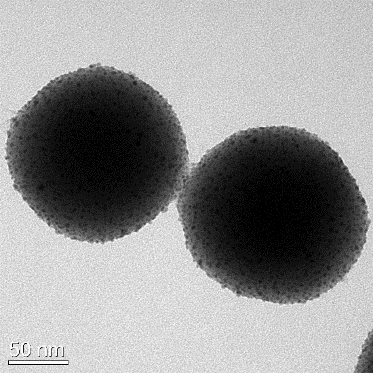

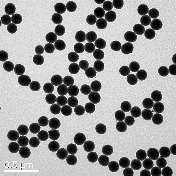


(a)

(b)

(c)

(d)


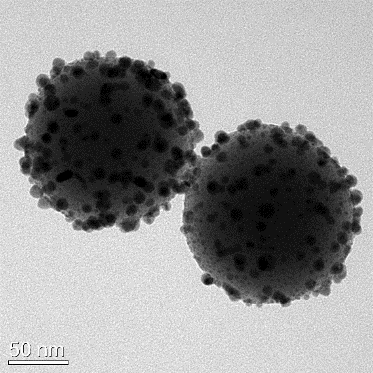

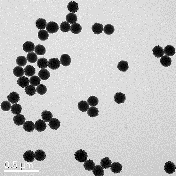

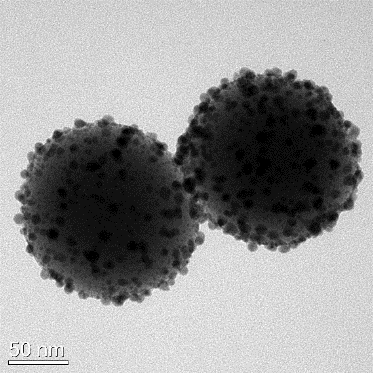

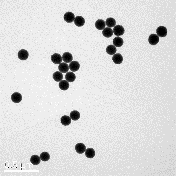

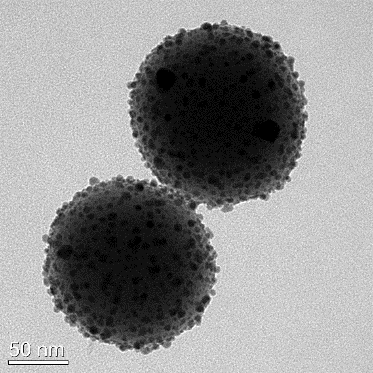

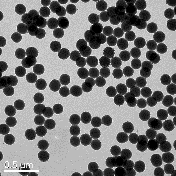

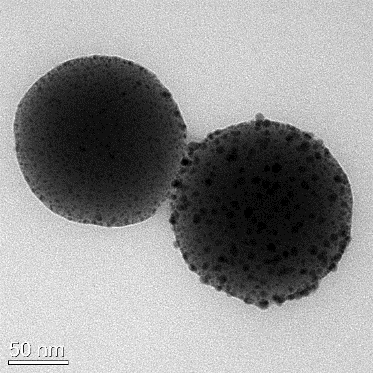

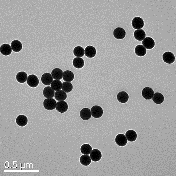

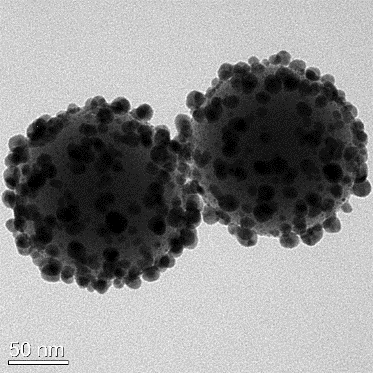

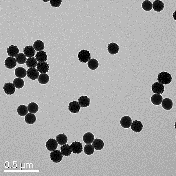

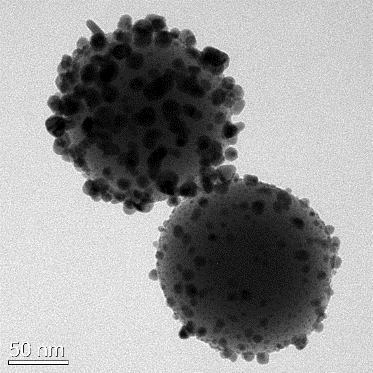

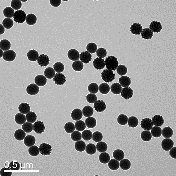

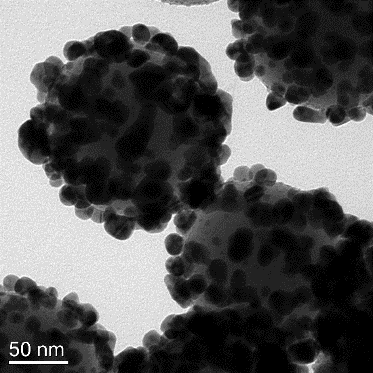

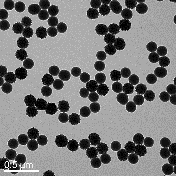


(e)

(f)

(g)

(h)

(i)

(k)

(l)

Figure S2. Transmission electron microscopy (TEM) images of SiO_2_@Au@Ag synthesized at different concentrations of ascorbic acid: (a) 0, (b) 2, (c) 4, (d) 6, (e) 8, (f) 10, (g) 20, (h) 40, (i) 60, (k) 80, (l) 100 µM. Measurement values of SiO_2_@Au and AgNO_3_ are 100 µg and 1 nmol, respectively.


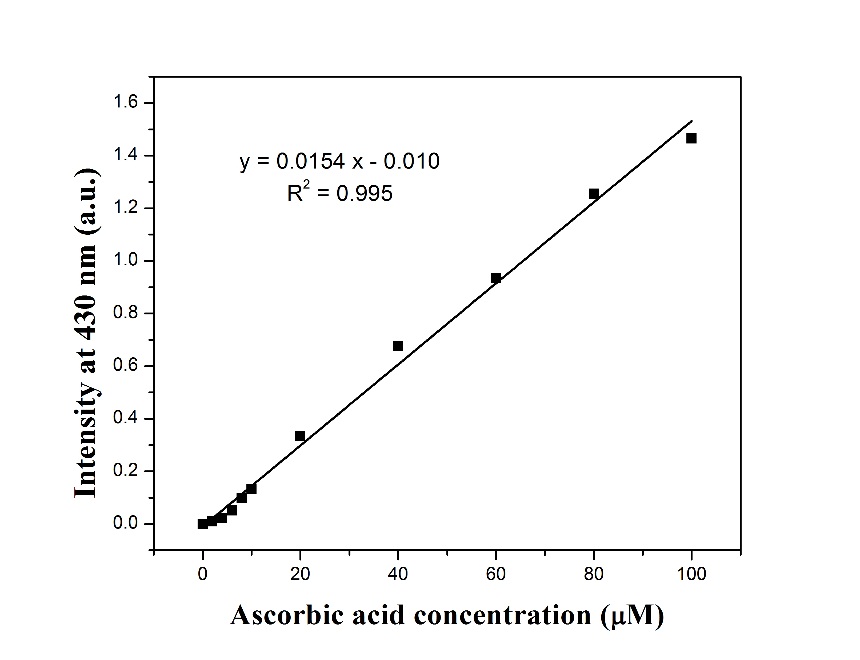

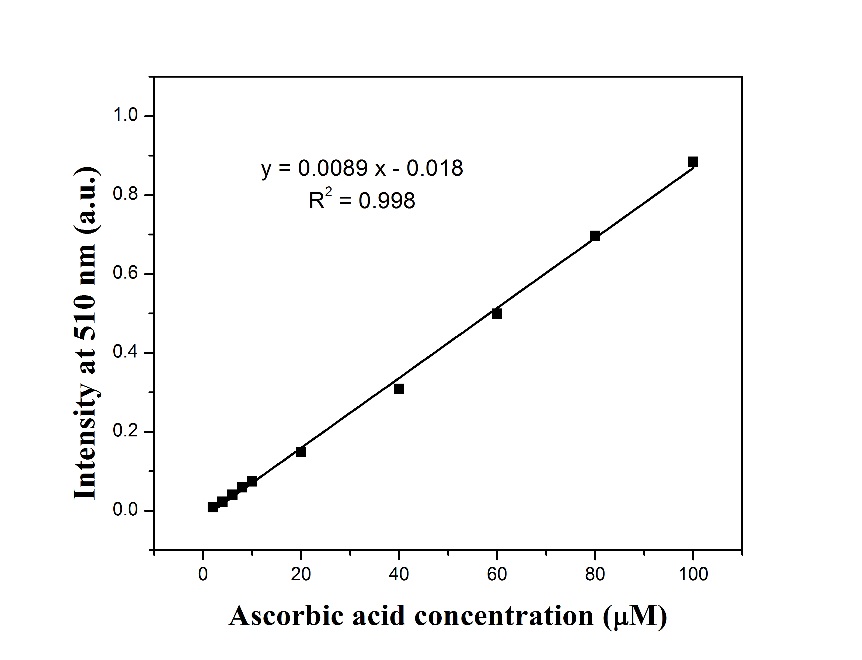


(a)

(b)

Figure S3. Calibration curves of SiO_2_@Au@Ag at (a) 430 nm and (b) 510 nm at various concentrations of ascorbic acid. Error bars show the standard deviation of three measurements.


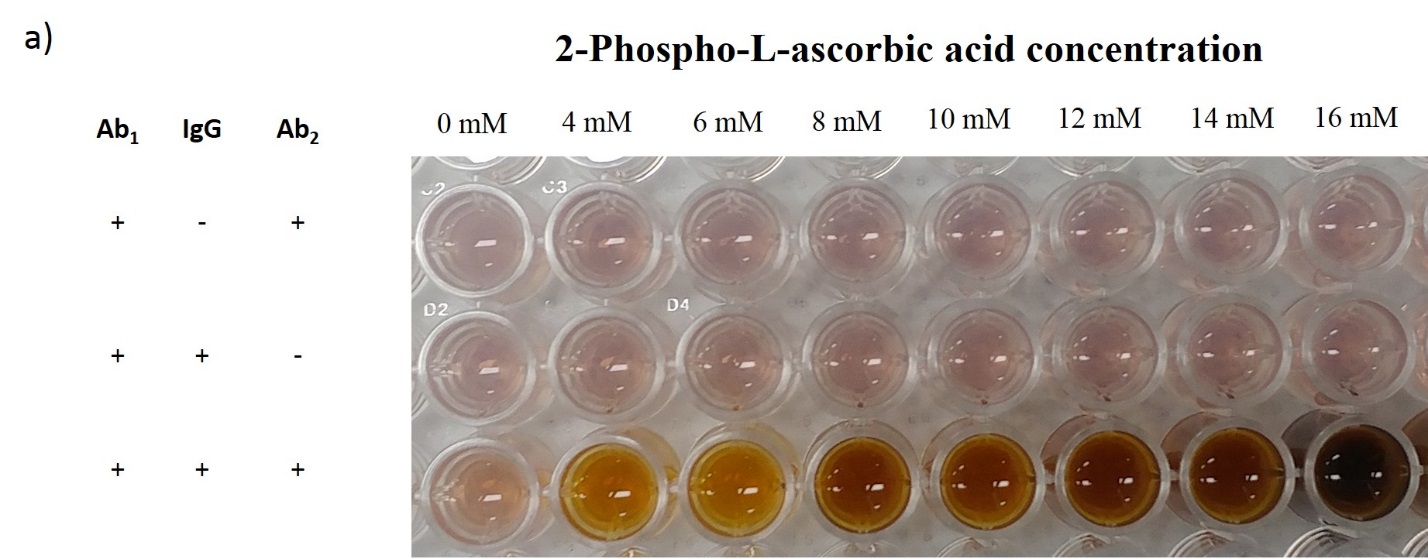


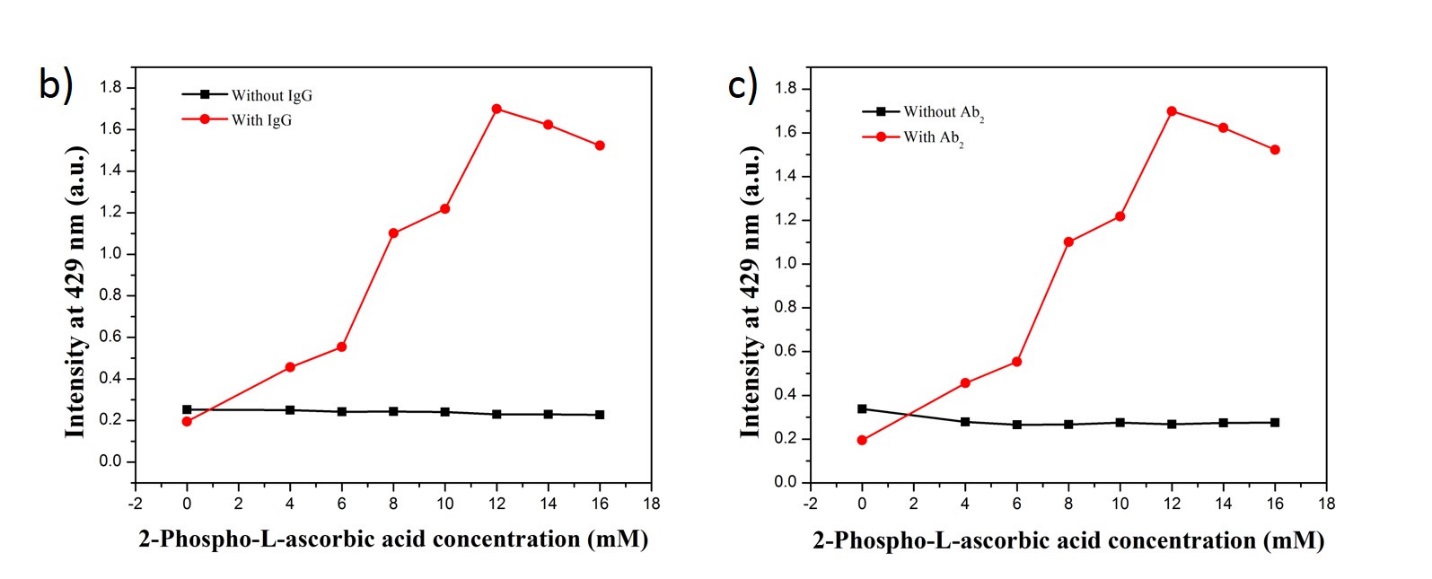


Figure S4. Effect of 2-phospho-L-acscorbic acid concentration on SiO2@Au@Ag growth in the presence of IgG and Ab2. a) Naked-eye detection of SiO_2_@Au@Ag with different concentration of 2-phospho-L-ascorbic acid (from 2 mM to 16 mM). (b) The UV-Vis spectra and the absorbance intensity curves of the SiO_2_@Au@Ag in the absence and presence of IgG. (c) The UV-Vis spectra and the absorbance intensity curves of the SiO_2_@Au@Ag in the absence and presence of Ab_2_. IgG (7 x 10^-9^ M), Ag^+^ (10 mM), and SiO_2_@Au (3 mg/mL) were used in this study.


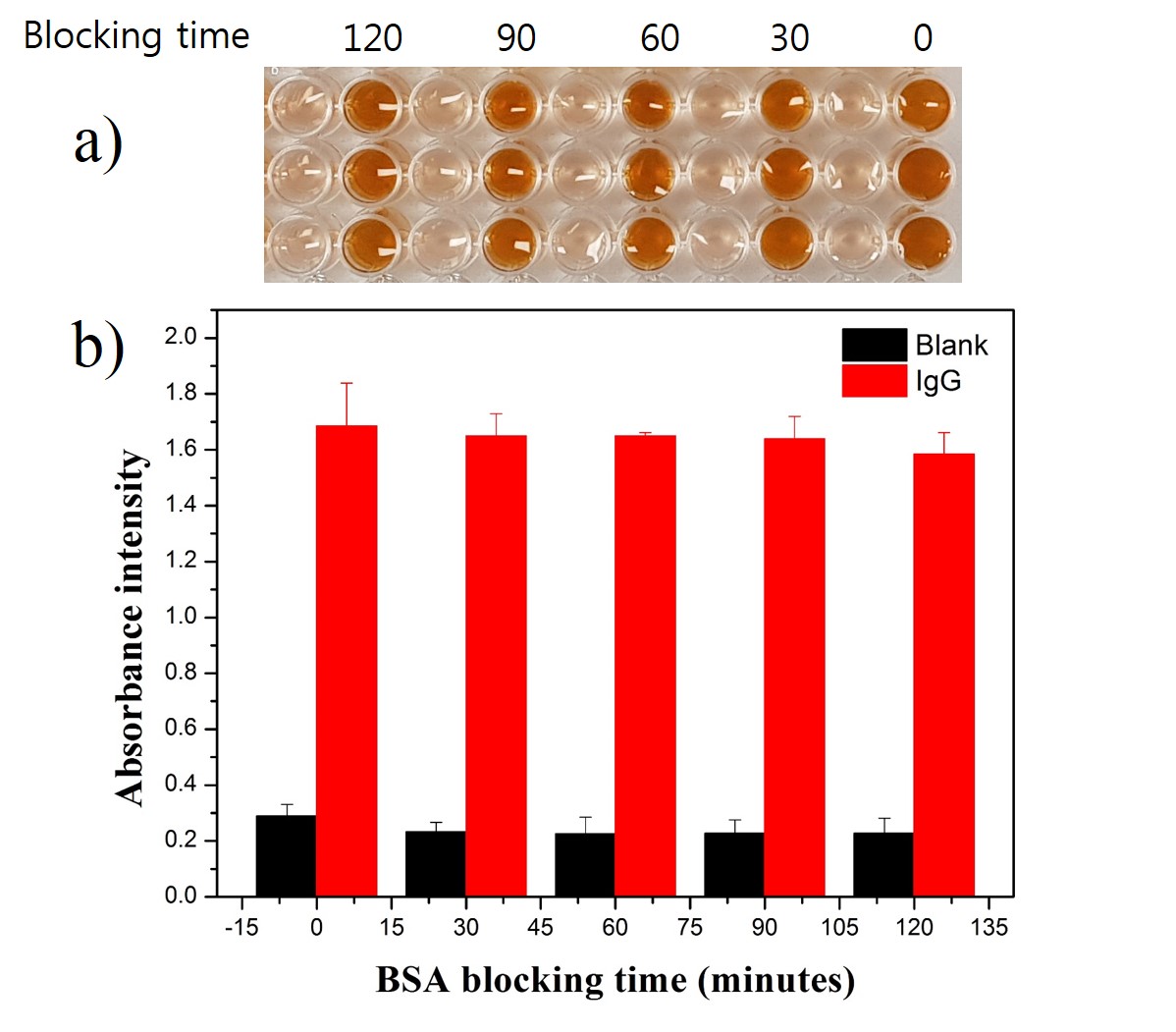


Figure S5. Effect of BSA blocking time on SiO_2_@Au@Ag growth. a) Naked-eye detection of SiO_2_@Au@Ag at different incubation time points (from 0 to 120 minutes), (b) intensity plot of SiO_2_@Au@Ag as a function of incubation time. IgG (7 x 10^-9^ M), Ag^+^ (10 mM), and SiO_2_@Au (3 mg/mL) were used in this study. Error bars show the standard deviations of three independent measurements.


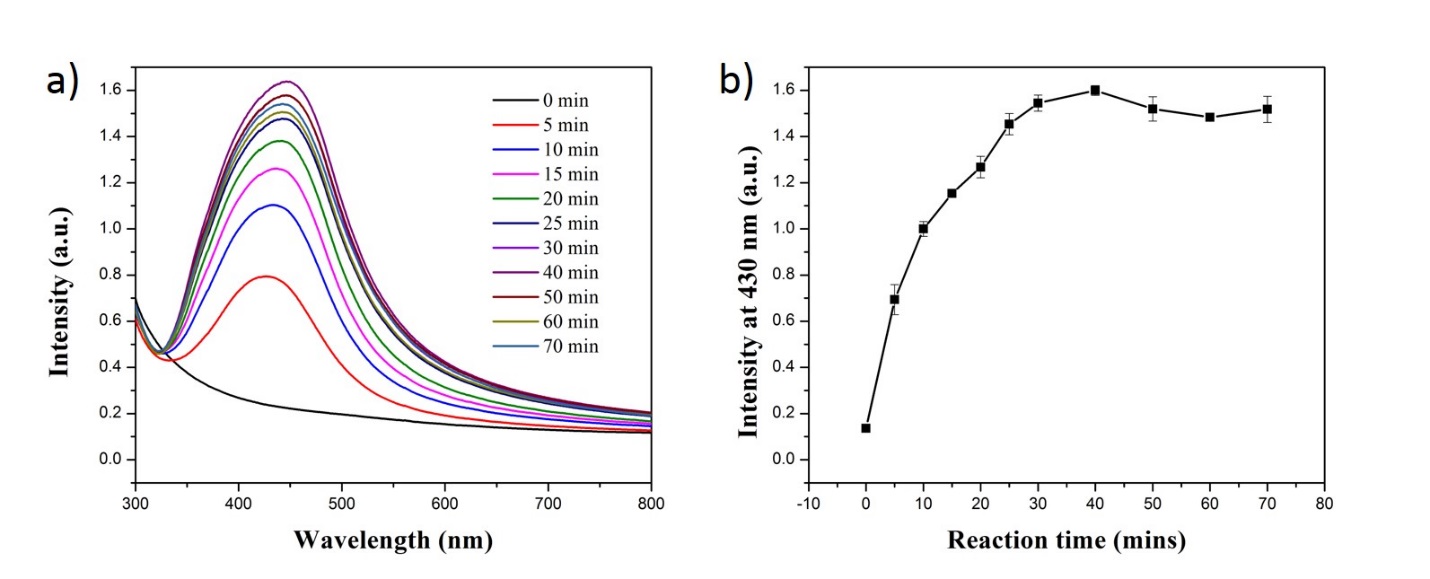


Figure S6. Effect of enzyme incubation time on SiO_2_@Au@Ag growth. a) UV-Vis spectra and (b) intensity plot of SiO_2_@Au@Ag as a function of incubation time. IgG (7 x 10^-9^ M), Ag^+^ (10 mM), and SiO_2_@Au (3 mg/mL) were used in this study. Error bars show the standard deviations of three independent measurements.


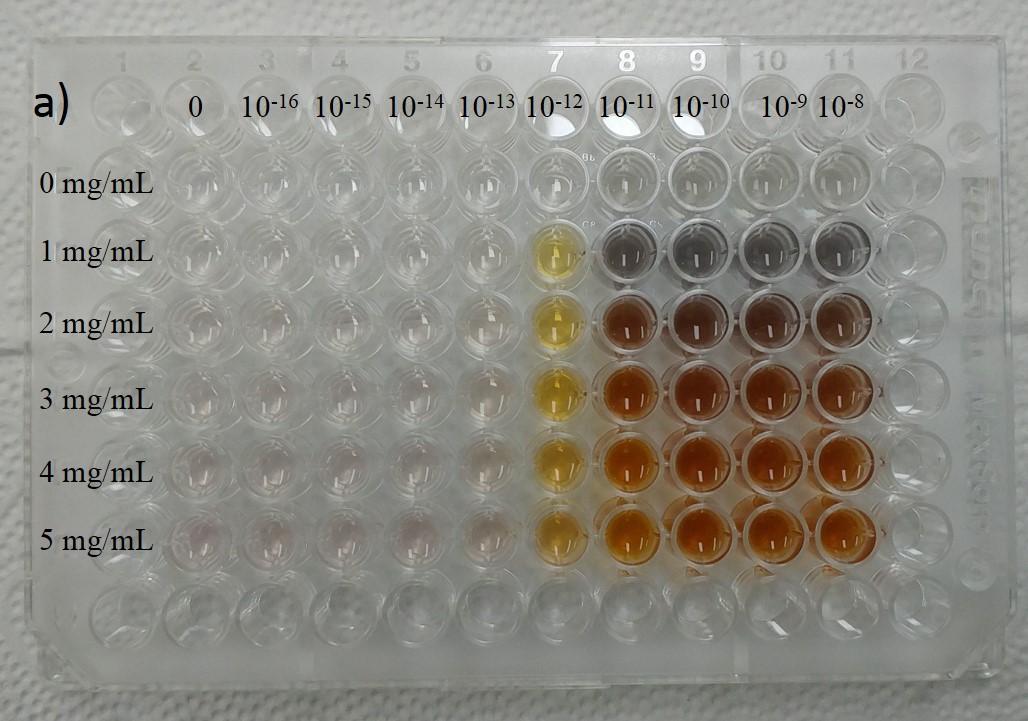

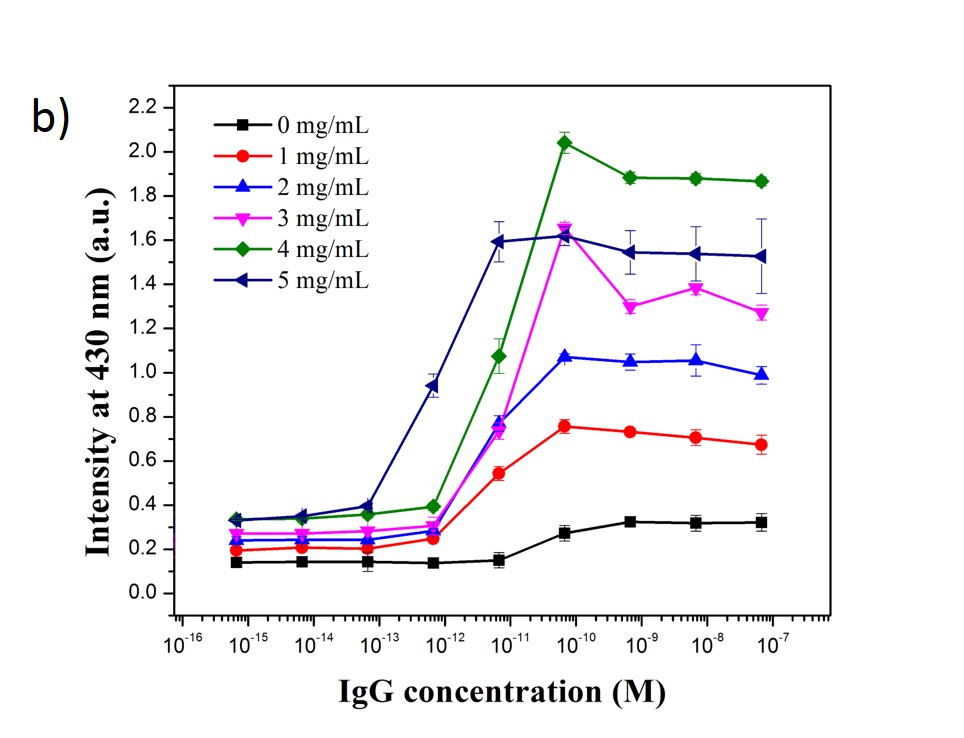


Figure S7. Effect of SiO_2_@Au loading amount on SiO_2_@Au@Ag growth. SiO_2_@Au concentration was in the range of 0−5 mg/mL. IgG (7 x 10^9^ M) and Ag^+^ (10 mM)were used in this study.. Error bars show the standard deviations of three independent measurements.


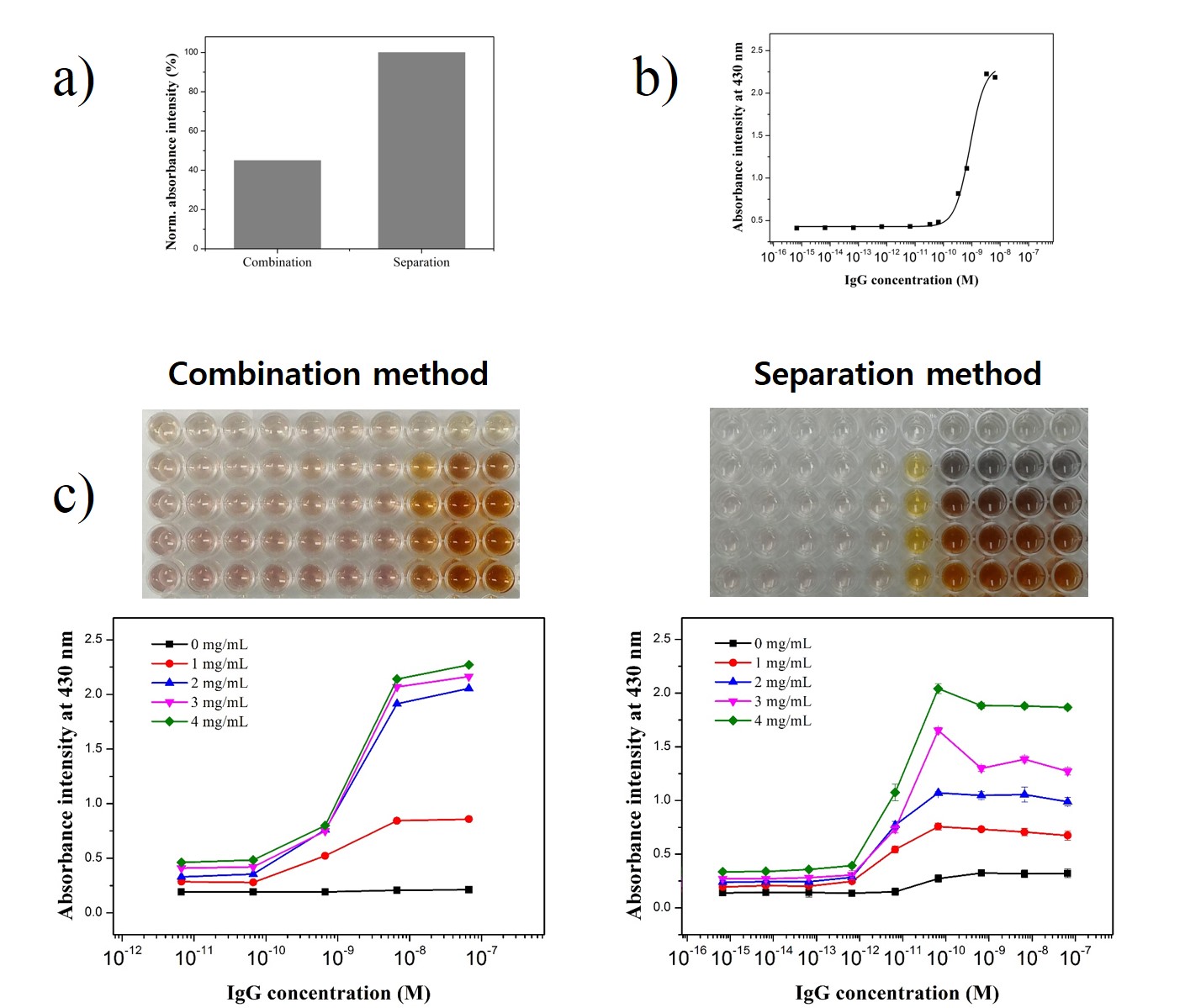


Figure S8. Comparison of the absorbance intensities of SiO_2_@Au@Ag prepared using the “combination” and “separation” methods (a) Normalized absorbance intensity of SiO_2_@Au@Ag produced by 4 × 10^-10^ M IgG. (b) Absorbance intensity plot of SiO_2_@Au@Ag produced by IgG in the ranged of 7 ×10^-16^ −7 × 10^-9^ M. (c) Effect of SiO_2_@Au loading amount on the growth of SiO_2_@Au@Ag produced by IgG in the range of 7 ×10^-16^ −7 × 10^-9^ M. The SiO_2_@Au concentration was in the range of 0−4 mg/mL. IgG (7 x 10^-9^ M) and Ag^+^ (10 mM) were used in this study. Error bars show the standard deviations of three independent measurements.
